# Gateways for Glutamate Neuroprotection in Parkinson’s Disease (PD): Essential Role of EAAT3 and NCX1 Revealed in an In Vitro Model of PD

**DOI:** 10.3390/cells9092037

**Published:** 2020-09-06

**Authors:** Silvia Piccirillo, Simona Magi, Alessandra Preziuso, Pasqualina Castaldo, Salvatore Amoroso, Vincenzo Lariccia

**Affiliations:** Department of Biomedical Sciences and Public Health, School of Medicine, University “Politecnica delle Marche”, Via Tronto 10/A, 60126 Ancona, Italy; s.piccirillo@staff.univpm.it (S.P.); s.magi@staff.univpm.it (S.M.); alessandra.preziuso@hotmail.it (A.P.); p.castaldo@staff.univpm.it (P.C.); s.amoroso@staff.univpm.it (S.A.)

**Keywords:** EAAT3, glutamate, mitochondrial dysfunction, Parkinson’s disease, NCX1, neuronal survival

## Abstract

Increasing evidence suggests that metabolic alterations may be etiologically linked to neurodegenerative disorders such as Parkinson’s disease (PD) and in particular empathizes the possibility of targeting mitochondrial dysfunctions to improve PD progression. Under different pathological conditions (i.e., cardiac and neuronal ischemia/reperfusion injury), we showed that supplementation of energetic substrates like glutamate exerts a protective role by preserving mitochondrial functions and enhancing ATP synthesis through a mechanism involving the Na^+^-dependent excitatory amino acid transporters (EAATs) and the Na^+^/Ca^2+^ exchanger (NCX). In this study, we investigated whether a similar approach aimed at promoting glutamate metabolism would be also beneficial against cell damage in an in vitro PD-like model. In retinoic acid (RA)-differentiated SH-SY5Y cells challenged with α-synuclein (α-syn) plus rotenone (Rot), glutamate significantly improved cell viability by increasing ATP levels, reducing oxidative damage and cytosolic and mitochondrial Ca^2+^ overload. Glutamate benefits were strikingly lost when either EAAT3 or NCX1 expression was knocked down by RNA silencing. Overall, our results open the possibility of targeting EAAT3/NCX1 functions to limit PD pathology by simultaneously favoring glutamate uptake and metabolic use in dopaminergic neurons.

## 1. Introduction

Parkinson’s disease (PD) is a neurodegenerative disorder characterized by the progressive loss of dopaminergic neurons in the substantia nigra pars compacta and the development of cytoplasmic aggregation of α-synuclein (α-syn), known as Lewy bodies [1,2,3,4]. The majority of PD cases (90–95%) are believed to be sporadic and most likely caused by a complex interaction between genetic susceptibility and environmental factors. The remaining 5–10% of the cases report different version of the familial forms of PD characterized by autosomal dominant or recessive monogenic mutations in several genes such as α-syn, LRRK2, Parkin, PINK and DJ-1 [4,5,6,7,8,9,10]. Although the sequential events leading to degeneration of dopaminergic neurons is still poorly defined, many lines of evidence highlight that mitochondrial dysfunction is a common pathological feature shared by both sporadic and monogenic PD [11,12,13,14,15,16,17]. The first evidence of mitochondrial involvement in the pathogenesis of PD comes from the seminal finding that electron transfer chain complex I deficiency, induced by the mitochondrial toxin 1-methyl-4-phenyl-1,2,3,6-tetrahydropyridine (MPTP), results in an acute and irreversible parkinsonian syndrome, characterized by the loss of bioenergetic function, free radicals overproduction and impaired Ca^2+^ homeostasis [18,19,20]. Respiratory chain dysfunctions, including mitochondrial complex I deficiency, were later documented in individuals with sporadic PD [12,21]. In addition, it has also been reported that reduced mitochondrial complex I activity is involved in the mechanisms leading to PD induced by oligomeric form of α-syn [22,23,24,25,26,27]. Furthermore, a direct association between mitochondrial defects and the development of PD arises from studies showing that other toxins, such as rotenone (Rot; an inhibitor of respiratory chain complex I) [3], can induce α-syn aggregation, leading to mitochondrial toxicity as well as the loss of dopaminergic neurons [22,25,28,29]. Finally, mutations in different PD-related genes can significantly affect core functions of mitochondria and ultimately disrupt mitochondrial homeostasis [30,31]. All the above observations have inspired the possibility to target mitochondria and cell energy metabolism to prevent and/or treat PD [32,33]. To date, only few studies have focused on the recovery of mitochondrial activity as a neuroprotective strategy to minimize neuronal loss (i.e., the use of alternative metabolic sources [32,34,35], the modulation of mitochondrial enzymes involved in glutamate metabolism [36] or the administration of a membrane-permeable prodrug of the complex II [37]).

We have previously demonstrated that in different cell models and experimental settings, glutamate supplementation stimulates ATP synthesis under pathophysiological conditions [38,39,40,41]. Remarkably, this glutamate-related response strictly relies on the activity of two main players: Na^+^-dependent excitatory amino acid transporters (EAATs) and the Na^+^/Ca^2+^ exchanger (NCX).

EAATs are electrogenic transport systems that couple the entry of 1 molecule of glutamate, 3 Na^+^ and 1 H^+^ ions, with the counter transport of 1 K^+^ ion [42,43]. NCX is a key player in controlling ionic homeostasis at both the plasma membrane and the mitochondrial levels, mediating Na^+^ influx/Ca^2+^ efflux (forward mode) or Na^+^ efflux/Ca^2+^ influx (reverse mode) [44,45]. Three NCX isoforms have been identified, namely NCX1, NCX2 and NCX3, which are expressed in a tissue- specific manner [45,46,47,48,49]. In this regard, we have previously demonstrated a physical and functional interaction between a specific EAATs subtype, EAAT3 (excitatory amino acid carrier 1, EAAC1 in rodents) and NCX1 at both the plasma membrane and the mitochondrial levels [38,41,50]. In both cardiac and neuronal models, their interaction modulates the glutamate-driven ATP synthesis under pathophysiological conditions [38,39,40,41,51].

On the basis of this evidence, we investigated the ability of glutamate to recover oxidative metabolism, cell viability and intracellular Ca^2+^ dysfunctions in an in vitro model of PD based on retinoic acid (RA)-differentiated SH-SY5Y neuroblastoma dopaminergic cells exposed to α-syn plus Rot treatment [22], and explored the possible involvement of NCX1 and EAAT3.

## 2. Materials and Methods

### 2.1. Cell Culture and Treatments

The human neuroblastoma cell line SH-SY5Y5 was obtained from American Type Culture Collection (CRL-2266). SH-SY5Y cells were cultured on 100 mm Petri dishes using Eagle’s minimum essential medium/nutrient mixture Ham’s F-12 (Corning, New York, USA) (1:1) media supplemented with 10% fetal bovine serum, 100 U/mL penicillin, and 100 μg/mL streptomycin. The cell culture medium was replaced every 2 days. The cells were maintained in a humidified incubator at 37 °C and 5% CO_2_. Differentiation into neuron-like cells was achieved by treatment with 10 μM all-trans RA [40]. Cells were challenged for 24 h with α-syn (10 nM) plus Rot (300 nM) and glutamate (500 µM) was added during the last hour of the treatment. Then cell viability, mitochondrial activity, ATP synthesis and mitochondrial reactive oxygen species (ROS) production were assessed. Cells were acutely exposed to α-syn (10 nM), Rot (300 nM) and glutamate (500 µM) to analyze cytoplasmic and mitochondrial Ca^2+^ levels.

### 2.2. Generation of HNE-Induced α-Syn Oligomers

Oligomers of α-syn were produced as described earlier [52] with minor modification. Briefly, monomeric α-syn human recombinant (140 μM) (Sigma-Aldrich, St. Louis, MO, USA) was incubated with 4-hydroxy-2-nonenal (HNE) (Santa Cruz Biotechnology, Dallas, TX, USA) in an HNE:α-syn molar ratio of 20:1 at 37 °C for 96 h. After incubation, unbound aldehyde was removed with an Amicon 3 KDa cut-off ultra-centrifugal unit (Millipore, Burlington, MA, USA).

### 2.3. Silencing of NCX1 and EAAT3

Silencing of NCX1 and EAAT3 RNA was performed as previously described [40,53]. Briefly, RA-differentiated SH-SY5Y cells were transfected with a HiPerfect Transfections Kit (Qiagen, Hilden, Germany) according to the manufacturer’s instruction by using FlexiTube small interfering RNA (siRNA) for NCX1 (Qiagen, Hs_SLC8A1_9, 5′-CAGGCCATCTTCTAAGACTGA-3′) and FlexiTube siRNA for EAAT3 (Qiagen, Hs_SLC1A1_3, 5′-GAGGACTGTTCTAACTAGTAA-3′).

Cells were harvested and analyzed by western blot for EAAT3 and NCX1 protein levels at 48 h after transfection [40]. The efficacy of the siRNA-based silencing was verified by assessing the protein level of targeted transporter, which in our settings was reduced by ~50% and by >70% for NCX1 and EAAT3, respectively [40].

At the end of the silencing protocol, cells were subjected to the specific treatment and then tested for cell viability, ATP content, ROS production and Ca^2+^ levels.

### 2.4. Cell Viability

Cell damage was evaluated by measuring the release of lactate dehydrogenase (LDH) into the culture supernatant. RA-differentiated SH-SY5Y were cultured on 12-well plates (7.5 × 10^4^ cells/well) and, after being collected and centrifugated (250× *g* 1 min), 100 µL of supernatants was incubated with the same volume of reaction mixture (Roche Diagnostics, Monza, Italy) for 30 min at room temperature in the dark. The released LDH from dying cells was measured by reading the absorbance at 490 nm in a Victor Multilabel Counter plate reader (Perkin Elmer, Waltham, MA, USA).

### 2.5. Mitochondrial Activity

Mitochondrial activity was measured by 3-(4,5-dimethylthiazol-2-yl)-2,5-diphenyltetrazolium bromide (MTT) assay, which is based on the reduction of MTT into insoluble formazan by the activity of mitochondrial enzymes [54]. Briefly, RA-differentiated SH-SY5Y were cultured on 12-well plates (7.5 × 10^4^ cells/well) and, at the end of the experimental protocol, cells were incubated with 1 mL of MTT reagent (0.5 mg/mL in PBS) to each well in the dark at 37 °C, in a humidified incubator, in a 5% CO_2_ atmosphere. After 1 h, the supernatant was removed and centrifuged to collect all the formazan crystals, which were dissolved in 1 mL of DMSO. Finally, the absorbance was measured at 540 nm using a Victor Multilabel Counter plate reader (Perkin Elmer). Cell viability was expressed as percentage of the control.

### 2.6. ATP Assay

Measurement of ATP synthesis was performed by using a commercially available luciferase-luciferin system (ATPlite, Perkin Elmer) according to the manufacturer’s instructions [40]. RA-differentiated SH-SY5Y cells were cultured on 96-well ViewPlate (Perkin Elmer) (1 × 10^5^ cells/well) and, after being subjected to the specific treatment, intracellular ATP levels were analyzed with a luminescence counter (Victor Multilabel Counter, Perkin Elmer), normalized to the respective protein content and expressed as percentage of the control.

### 2.7. Detection of Mitochondrial ROS Formation

Mitochondrial ROS production was evaluated using the mitochondrial-targeted dye Mitotracker CM-H2XRos [55] (Invitrogen Life Technologies, Carlsbad, CA, USA). RA-differentiated cells were cultured on glass coverslip on 6-well plates (1 × 10^5^ cells/well) and, after being treated, cells were loaded with 300 nM of the dye for 30 min in the dark at 37 °C. For live cell imaging of mitochondrial ROS, measurements were conducted on the confocal microscope Zeiss 510 LSM (Carl Zeiss, Milan, Italy). CM-H2XRos was excited with 561 nm laser, and emission was detected above 580 nm and recorded for 120 s. The analysis of red fluorescence increase was performed off-line after images acquisition. Fluorescence values were reported as percentage of the control.

### 2.8. Analysis of Cytoplasmic and Mitochondrial Ca^2+^ Levels

Cytoplasmic and mitochondrial Ca^2+^ levels were monitored by single cell computer-assisted video imaging using LSM 510 confocal system (Carl Zeiss), as described previously [22]. Briefly, RA-differentiated SH-SY5Y cell were loaded with 5 µM Rhod 2-AM (Abcam, Cambridge, UK) in DMEM medium for 15 min in the dark at 37 °C; then, 4 μM Fluo-4/AM (Invitrogen Life Technologies) and 0.08% pluronic acid (Molecular Probe, Eugene, OR, USA) were added to the medium for further 45 min. Coverslips were then washed twice in PBS, placed into a perfusion chamber mounted onto the stage of an inverted Zeiss Axiovert 200 microscope, and maintained in standard buffer solution (in mM: 140 NaCl, 5 KCl, 1 CaCl_2_, 0.5 MgCl_2_, 10 HEPES, 5.5 glucose, buffered to pH 7.4 with NaOH) at 37 °C using a heated microscope stage and climate box from PeCon GmbH. Both cytoplasmic and mitochondrial Ca^2+^ elevations were evaluated as fluorescence increase. Bath solution was changed with a peristaltic pump and images were acquired every 5 s. Fluo 4-AM was excited at 488 nm and the emission was time-lapsed recorded at 505–530, while Rhod 2-AM was excited at 543 nm and fluorescence emission was measured from 560 nm to 600 nm. Analysis of fluorescence was performed off-line after images acquisition, as previously described [22].

### 2.9. Drug and Chemicals

SN-6, 2-[[4-[(4Nitrophenyl) methoxy] phenyl] methyl]-4-thiazolidinecarboxylic acid ethyl ester (SN-6) and DL-threo-beta-benzyloxyaspartate (DL-TBOA) were obtained from Tocris. All the other chemicals were of analytical grade and were purchased from Sigma.

### 2.10. Data Analysis

Data were expressed as mean ± S.E.M. Values less than 0.05 were considered to be significant. Differences among means were assessed by one-way ANOVA followed by Dunnet’s post hoc test. Statistical comparisons were carried out using the GraphPad Prism 5 software (GraphPad Software Inc., San Diego, CA, USA). Concentration–effect curves were obtained by fitting the data to the four-parameter logistic equation y = min + (max − min)/(1 + (x/EC50)n), where y is the variable of interest (normalized LDH or MTT signals), x is the α-syn concentration in nmoles/L, and n is the Hill coefficient. Data fitting was performed using the fitting routines of the Origin 8.0 software (OriginLab, Northampton, MA, USA).

## 3. Results

### 3.1. Effect of α-Syn and α-Syn Plus Rot on Cell Viability

**Figure 1:**
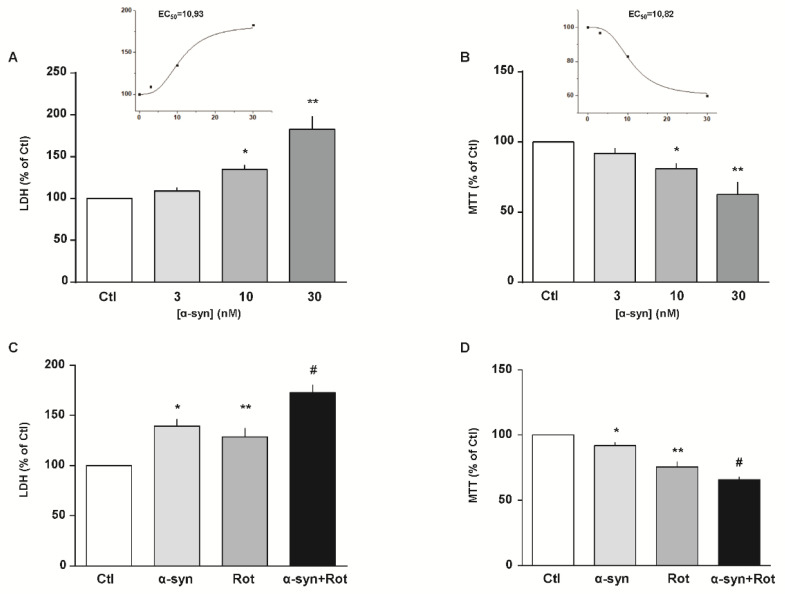
Effect of α-syn and α-syn plus Rot on cell survival. (**A**–**D**) Cell injury and mitochondrial activity induced by 24 h exposure to increasing concentrations of α-syn (from 3 to 30 nM) and (**A**,**B**) α-syn (10 nM) plus Rot (300 nM) (**C**,**D**) were assessed by means of lactate dehydrogenase (LDH) (**A**,**C**) and 3-(4,5-dimethylthiazol-2-yl)-2,5-diphenyltetrazolium bromide (MTT) assays (**B**,**D**). Extracellular LDH release and MTT reduction were expressed as percentage of the control. In each panel, the inset shows the EC50 values of α-syn estimated from each specific assay. Statistical differences were assessed by one-way ANOVA followed by Dunnet’s post hoc test. (**A**) F (3,22) = 25.10. Each column represents the mean ± S.E.M. of at least n = 5 independent experiments performed in triplicate. * Significant versus all groups (*p* < 0.01 versus Ctl, *p* < 0.05 versus 3 nM, *p* < 0.001 versus 30 nM); ** significant versus all groups (*p* < 0.0001 versus Ctl and 3 nM, *p* < 0.001 versus 10 nM). (**B**) F (3,36) = 21.16. Each column represents the mean ± S.E.M. of at least n = 8 independent experiments performed in triplicate. * Significant versus all groups (*p* < 0.01 versus Ctl and 30 nM, *p* < 0.05 versus 3 nM); ** significant versus all groups (*p* < 0.0001 versus Ctl and 3 nM, *p* < 0.01 versus 10 nM). (**C**) F (3,12) = 19.85. Each column represents the mean ± S.E.M. of n = 4 independent experiments performed in triplicate. * Significant versus Ctl (*p* < 0.01) and α-syn+Rot (*p* < 0.05); ** significant versus Ctl (*p* < 0.05) and α-syn+Rot (*p* < 0.01); # significant versus all groups (*p* < 0.0001 versus Ctl, *p* < 0.05 versus α-syn, *p* < 0.01 versus Rot). (**D**) F (3,18) = 69.39 each column represents the mean ± S.E.M. of at least n = 4 independent experiments performed in triplicate. * Significant versus all groups (*p* < 0.05 versus Ctl, *p* < 0.001 versus Rot, *p* < 0.0001 versus α-syn+Rot); ** significant versus all groups (*p* < 0.0001 versus Ctl, *p* < 0.001 versus α-syn, *p* < 0.05 versus α-syn+Rot); # Significant versus all groups (*p* < 0.0001 versus Ctl and α-syn, *p* < 0.05 versus Rot). Ctl = control; α-syn = α-synuclein; Rot = rotenone.

We initially exposed RA-differentiated SH-SY5Y cells to increasing concentrations of α-syn (from 3 to 30 nM) for 24 h. As shown in Figure 1A,B, we found a concentration-dependent cytotoxic effect of α-syn on both cell injury and mitochondrial activity, as assessed by LDH and MTT assays, respectively. Based on these results, we chose 10 nM as the α-syn working concentration for the following experimental sets.

Since Rot by interacting with α-syn stimulates its aggregation and toxicity [28,29,56,57], we evaluated cell viability after exposure to α-syn plus Rot. In particular, we found that cell damage induced by syn (10 nM) plus Rot (300 nM) [22] was significantly higher than their respective single exposure (Figure 1C,D), and this combination of α-syn plus Rot was used to set up our in vitro PD-like model.

### 3.2. Glutamate Recovery of Cell Injury, ROS Overproduction and ATP Synthesis Reduction Induced by α-Syn Plus Rot

**Figure 2:**
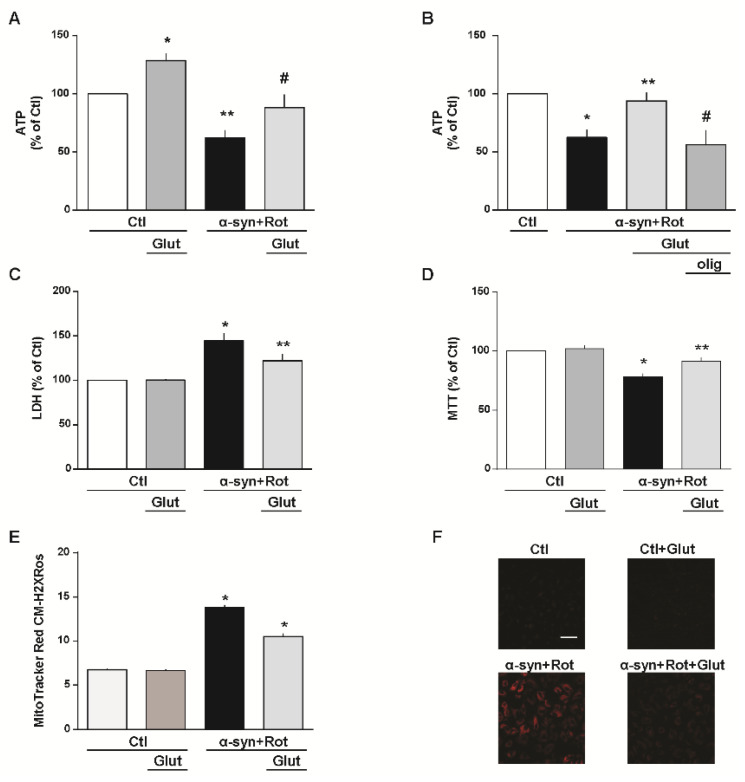
Glutamate recovery of cell injury, reactive oxygen species (ROS) overproduction and ATP synthesis reduction induced by α-syn plus Rot. Effect of 1 h exposure to glutamate under physiological conditions on ATP synthesis (**A**), extracellular LDH release (**C**), mitochondrial activity (**D**) and mitochondrial ROS production (**E**). A,B Intracellular ATP levels after 24 h exposure to α-syn (10 nM) plus Rot (300 nM) and 23 h exposure to α-syn (10 nM) plus Rot (300 nM) followed by 1 h exposure to glutamate (500 µM) in the absence (**A**) or in the presence of oligomycin (3 µg/mL) (**B**). In each experiment, ATP levels were normalized to the respective protein content and expressed as percentage of the control. (**C**,**D**) Cell injury, assessed by means of extracellular LDH and MTT assays, and (**E**,**F**) mitochondrial ROS production, assessed by measuring MitoTracker Red CM-H2XRos fluorescence intensity, were evaluated after 24 h exposure to α-syn (10 nM) plus Rot (300 nM), in the presence or in the absence of glutamate. Where indicated, glutamate (500 µM) was added during the last hour of the α-syn+Rot treatment. In each experiment, extracellular LDH release and MTT reduction were expressed as percentage of the control. (**F**) Representative images of mitochondrial ROS by MitoTracker Red CM-H2XRos staining. Images are representative of n = 4 independent experiments. Scale bar = 50 µm. Statistical differences were assessed by one-way ANOVA followed by Dunnet’s post hoc test. (**A**) F (3,20) = 14.85. Each column represents the mean ± S.E.M. of n = 6 independent experiments performed in triplicate. * Significant versus all groups (*p* < 0.05 versus Ctl, *p* < 0.0001 versus α-syn+Rot, *p* < 0.01 versus α-syn+Rot+Glut); ** significant versus all groups (*p* < 0.01 versus Ctl, *p* < 0.0001 versus Ctl+Glut, *p* < 0.05 versus α-syn+Rot+Glut); # significant versus Ctl+Glut (0.01) and α-syn+Rot (0.05). (**B**) F (3,12) = 7.902. Each column represents the mean ± S.E.M. of n = 4 independent experiments performed in triplicate. * Significant versus Ctl and α-syn+Rot+Glut (*p* < 0.05); * significant versus α-syn+Rot and α-syn+Rot+Glut+olig (*p* < 0.05); # significant versus Ctl (*p* < 0.01) and α-syn+Rot+Glut (*p* < 0.05). (**C**) F (3,37) = 13.64. Each column represents the mean ± S.E.M. of at least n = 8 independent experiments performed in triplicate. * Significant versus all groups (*p* < 0.0001 versus control groups, *p* < 0.05 versus α-syn+Rot+Glut); ** significant versus Ctl and α-syn+Rot (*p* < 0.05). (**D**) F (3,17) = 19.37. Each column represents the mean ± S.E.M. of at least n = 3 independent experiments performed in triplicate. * Significant versus all groups (*p* < 0.0001 versus control groups, *p* < 0.01 versus α-syn+Rot+Glut); ** significant versus Ctl+Glut (*p* < 0.05) and α-syn+Rot (*p* < 0.01). (**E**) F (3,1714) = 188.9. The bar plot reports the mean ± S.E.M. of fluorescence increase elicited by ROS formation. For each experimental group, basal values used for the statistical analysis derived from n = 4 independent experiments, and 100–150 cells were recorded for each session. * Significant versus all groups (*p* < 0.0001). Olig = oligomycin.

Numerous studies showed that mitochondrial dysfunction and oxidative damage, whether triggered by environmental and/or genetic factors, contribute to the cascade of events leading to the PD neurodegeneration [21,58]. Firstly, we tested whether glutamate, as metabolic substrate, may attenuate cell damage induced by α-syn plus Rot. As expected, in RA-differentiated SH-SY5Y cells, glutamate significantly increased physiological ATP synthesis, confirming our previous results obtained in other cell models and different experimental settings [38,39,40,41] (Figure 2A). After α-syn plus Rot treatment, glutamate administration evoked an increase in ATP production up to the levels observed under control conditions (Figure 2A). Considering that 3 μg/mL of oligomycin, an ATP synthase inhibitor [40,41], counteracted the intracellular ATP increase induced by glutamate (Figure 2B), we suggest that this metabolic substrate may promote ATP synthesis by refilling the tricarboxylic acid (TCA) cycle and oxidative phosphorylation [39,40,41]. Furthermore, glutamate, which alone did not display any toxic effect, strongly prevented cell injury induced by α-syn plus Rot treatment (Figure 2C,D).

After investigating the ability of glutamate to stimulate the synthesis of ATP, we analyzed whether this amino acid modified ROS levels in our model. Effectively, as shown in Figure 2E,F, we found that glutamate partially prevented ROS overproduction induced by α-syn plus Rot.

### 3.3. Involvement of EAAT3 and NCX1 in the Glutamate Neuroprotection Against α-Syn Plus Rot Toxicity

**Figure 3:**
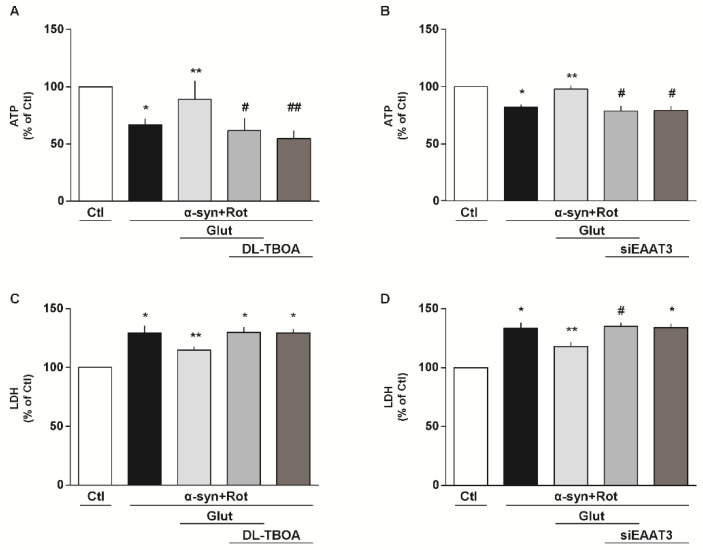
Involvement of EAATs in the glutamate neuroprotection against α-syn plus Rot toxicity. (**A**,**C**) Intracellular ATP content (**A**) and extracellular LDH (**C**) after 24 h exposure to α-syn (10 nM) plus Rot (300 nM) and 23 h exposure to α-syn (10 nM) plus Rot (300 nM) followed by 1 h exposure to glutamate (500 µM) in the presence or in the absence of DL-threo-beta-benzyloxyaspartate (DL-TBOA; 300 µM). (**B**,**D**) After 48 h of EAAT3 silencing, ATP levels (**B**) and cell damage (**D**) were evaluated after 24 h exposure to α-syn (10 nM) plus Rot (300 nM) and 23 h exposure to α-syn (10 nM) plus Rot (300 nM) followed by 1 h exposure to glutamate (500 µM). Statistical differences were assessed by one-way ANOVA followed by Dunnet’s post hoc test. (**A**) F (4,15) = 13.75. Each column represents the mean ± S.E.M. of n = 4 independent experiments performed in triplicate. * Significant versus Ctl (*p* < 0.01) and α-syn+Rot+Glut (*p* < 0.05); ** significant versus α-syn+Rot (*p* < 0.05), α-syn+Rot+Glut+DL-TBOA and α-syn+Rot+DL-TBOA (*p* < 0.01); # significant versus Ctl (*p* < 0.001) and α-syn+Rot+Glut (*p* < 0.01); ## significant versus Ctl (*p* < 0.0001) and α-syn+Rot+Glut (*p* < 0.01). (**B**) F (4,15) = 12.25. Each column represents the mean ± S.E.M. of n = 4 independent experiments performed in triplicate. * Significant versus Ctl and α-syn+Rot+Glut (*p* < 0.001); ** significant versus α-syn+Rot, α-syn+Rot+Glut+siEAAT3 and α-syn+Rot+siEAAT3 (*p* < 0.001); # significant versus Ctl (*p* < 0.0001) and α-syn+Rot+Glut (*p* < 0.001). (**C**) F (4,25) = 11.58. Each column represents the mean ± S.E.M. of n = 6 independent experiments performed in triplicate. * Significant versus Ctl (*p* < 0.0001) and α-syn+Rot+Glut (*p* < 0.05); ** significant versus all groups (*p* < 0.05). (**D**) F (4,10) = 20.73. Each column represents the mean ± S.E.M. of n = 3 independent experiments performed in triplicate. * Significant versus Ctl (*p* < 0.001) and α-syn+Rot+Glut (*p* < 0.05); ** Significant versus all groups (*p* < 0.05); # Significant versus Ctl (*p* < 0.0001) and α-syn+Rot+Glut (*p* < 0.05). siEAAT3 = siRNA for EAAT3.

**Figure 4:**
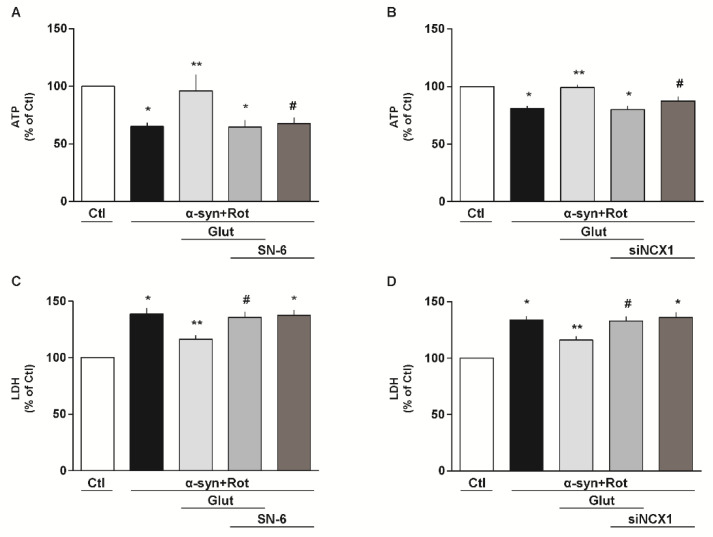
Involvement of NCX in the glutamate neuroprotection against α-syn plus Rot toxicity. (**A**,**C**) Intracellular ATP content (**A**) and extracellular LDH (**C**) after 24 h exposure to α-syn (10 nM) plus Rot (300 nM) and 23 h exposure to α-syn (10 nM) plus Rot (300 nM) followed by 1 h exposure to glutamate (500 µM) in the presence or in the absence of SN-6 (1 µM). (**B**,**D**) After 48 h of NCX1 silencing, ATP levels (**B**) and cell damage (**D**) were evaluated after 24 h exposure to α-syn (10 nM) plus Rot (300 nM) and 23 h exposure to α-syn (10 nM) plus Rot (300 nM) followed by 1 h exposure to glutamate (500 µM). The experiments reported in panel D were conducted simultaneously with the experiments reported in panel 3D. Statistical differences were assessed by one-way ANOVA followed by Dunnet’s post hoc test. (**A**) F (4,30) = 5.737. Each column represents the mean ± S.E.M. of n = 7 independent experiments performed in triplicate. * Significant versus Ctl (*p* < 0.001) and α-syn+Rot+Glut (*p* < 0.01); ** significant versus α-syn+Rot, α-syn+Rot+Glut+SN-6 and α-syn+Rot+SN-6 (*p* < 0.01); # significant versus Ctl and α-syn+Rot+Glut (*p* < 0.01). (**B**) F (4,20) = 13.62. Each column represents the mean ± S.E.M. of n = 5 independent experiments performed in triplicate. * Significant versus Ctl and α-syn+Rot+Glut (*p* < 0.001); ** significant versus α-syn+Rot, α-syn+Rot+Glut+siNCX1 (*p* < 0.001) and α-syn+Rot+siNCX1 (*p* < 0.05); # significant versus Ctl and α-syn+Rot+Glut (*p* < 0.05). (**C**) F (4,15) = 17.71. Each column represents the mean ± S.E.M. of n = 4 independent experiments performed in triplicate. * Significant versus Ctl (*p* < 0.0001) and α-syn+Rot+Glut (*p* < 0.01); ** significant versus all groups (*p* < 0.05 versus Ctl and α-syn+Rot+Glut+SN-6, *p* < 0.01 versus α-syn+Rot and α-syn+Rot+SN-6); # significant versus Ctl (*p* < 0.0001) and α-syn+Rot+Glut (*p* < 0.05). (**D**) F (4,15) = 19.99. Each column represents the mean ±  S.E.M. of n = 4 independent experiments performed in triplicate. * Significant versus Ctl (*p* < 0.0001) and α-syn+Rot+Glut (*p* < 0.01); ** significant versus all groups (*p* < 0.05 versus Ctl and α-syn+Rot+Glut+siNCX1, *p* < 0.01 versus α-syn+Rot and α-syn+Rot+siNCX1); # significant versus Ctl (*p* < 0.0001) and α-syn+Rot+Glut (*p* < 0.05). siNCX1 = siRNA for NCX1.

To investigate the involvement of EAATs and NCX in our experimental setting, we used both pharmacological and RNA interference (RNAi) approaches. When cells were exposed to the non-transportable EAAT blocker DL-threo-β-benzyloxyaspartic acid (DL-TBOA, 300 µM) [59] (Figure 3A,C) or to the selective NCX inhibitor 2-[[4-[(4Nitrophenyl) methoxy] phenyl] methyl]-4-thiazolidinecarboxylic acid ethyl ester (SN-6, 1 μM) [40,60] (Figure 4A,C), glutamate lost its protective effects in promoting ATP synthesis and cell survival. By using the siRNA approach, we verified that EAAT3 (Figure 3B,D) and NCX1 (Figure 4B,D) were specifically involved in the glutamate response against α-syn plus Rot-induced neurotoxicity.

### 3.4. Effect of Glutamate on α-Syn Plus Rot-Induced Cytoplasmic and Mitochondrial Ca^2+^ Increase: The Central Role of EAAT3 and NCX1

**Figure 5:**
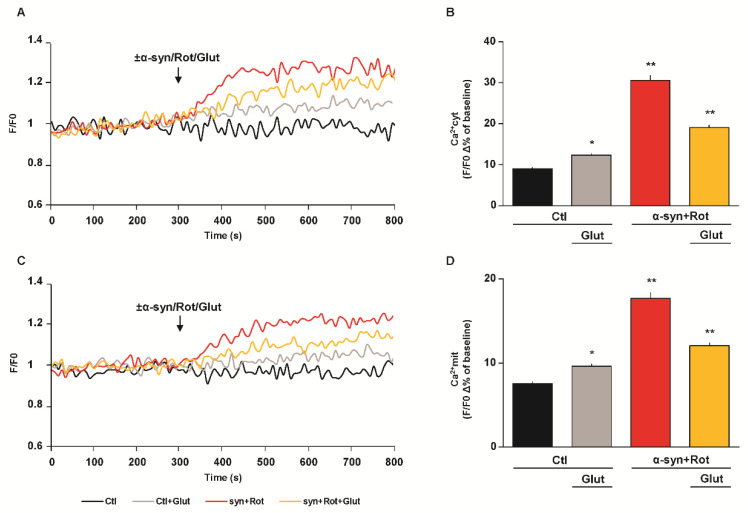
Effect of glutamate on α-syn plus Rot induced cytoplasmic and mitochondrial Ca^2+^ increase. (**A**,**C**) Representative records of cytoplasmic (**A**) and mitochondrial (**C**) Ca^2+^ levels under control conditions (black line), during acute exposure to glutamate (500 µM) (grey line), to α-syn (10 nM) plus Rot (300 nM) in the presence (orange line) or in the absence (red line) of glutamate. Fluorescence intensity was expressed as F/F0 ratio, where F is the background subtracted fluorescence intensity and F0 is the background subtracted mean fluorescence value measured from each cell at resting conditions (F/F0). (**B**,**D**) The bar plots showing cytoplasmic (**B**) and mitochondrial (**D**) Ca^2+^ levels depict the mean ± S.E.M of each Δ% fluorescence increase. For Δ% calculation, we used the maximal value of fluorescence obtained after stimulation and, as baseline, the mean of fluorescence recorded during the 300 s preceding the ±α-syn/Rot/Glut challenge. Statistical differences were assessed by one-way ANOVA followed by Dunnet’s post hoc test. (**B**) F (3,1118) = 122.2. For each experimental group, Δ% values used for the statistical analysis derived from 4 independent experiments and 50–100 cells were recorded in each different session. * Significant versus all groups (*p* < 0.05 versus Ctl, *p* < 0.0001 versus α-syn+Rot and α-syn+Rot+Glut); ** significant versus all groups (*p* < 0.0001). (**D**) F (3,1112) = 82.28. For each experimental group, Δ% values used for the statistical analysis derived from 5 independent experiments and 50–100 cells were recorded in each different session. * Significant versus all groups (*p* < 0.05 versus Ctl, *p* < 0.0001 versus α-syn+Rot and α-syn+Rot+Glut); ** significant versus all groups (*p* < 0.0001).

Both α-syn and Rot may compromise Ca^2+^ homeostasis by promoting intracellular Ca^2+^ overload, which consequently affects neuronal viability [25,61,62,63,64]. Therefore, we evaluated the effect of α-syn plus Rot on both cytoplasmic and mitochondrial Ca^2+^ levels and the effect of glutamate in our experimental model. We found that, in Fluo 4-AM and Rhod 2-AM loaded RA-differentiated cells, α-syn plus Rot acute exposure led to a significant rise of both cytoplasmic (Figure 5A,B) and mitochondrial Ca^2+^ levels (Figure 5C,D). As previously reported, under resting conditions [38,41], glutamate exposure induced a slight but significant elevation of both cytoplasmic (Figure 5A,B) and mitochondrial Ca^2+^ levels (Figure 5C,D). Furthermore, glutamate supplementation significantly reduced both cytoplasmic (Figure 5A,B) and mitochondrial Ca^2+^ increases (Figure 5C,D) induced by acute exposure to α-syn plus Rot.

**Figure 6:**
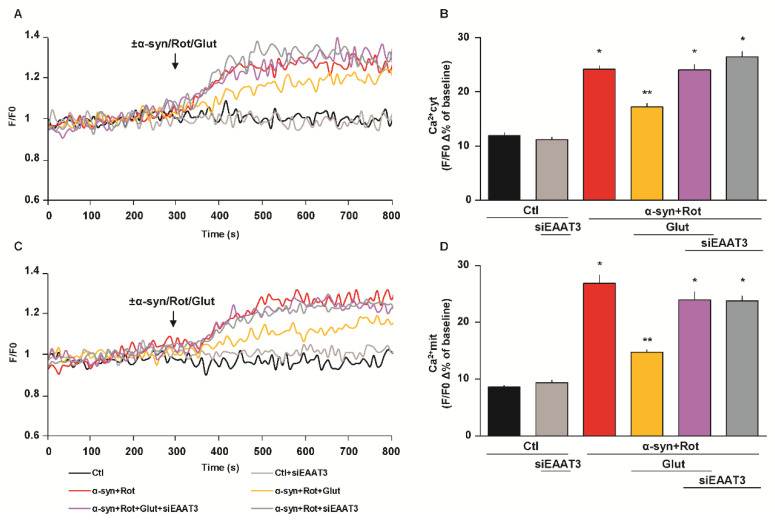
Effect of siEAAT3 on glutamate-induced reduction of cytoplasmic and mitochondrial Ca^2+^ levels. (**A**,**C**) Representative records of cytoplasmic (**A**) and mitochondrial (**C**) Ca^2+^ levels under control conditions (black line), after 48 h of EAAT3 silencing (light grey line), during acute treatment of α-syn (10 nM) plus Rot (300 nM) in the presence (grey line) or in the absence (red line) of siEAAT3, and acute treatment of α-syn plus Rot and glutamate (500 µM) in the presence (violet line) or in the absence (orange line) of siEAAT3. Fluorescence intensity was expressed as F/F0 ratio, where F is the background subtracted fluorescence intensity and F0 is the background subtracted mean fluorescence value measured from each cell at resting conditions (F/F0). (**B**,**D**) The bar plots showing cytoplasmic (**B**) and mitochondrial (**D**) Ca^2+^ levels depict the mean ± S.E.M of each Δ% fluorescence increase. For Δ% calculation, we used the maximal value of fluorescence obtained after stimulation and, as baseline, the mean of fluorescence recorded during the 300 s preceding the ±α-syn/Rot/Glut challenge. Statistical differences were assessed by one-way ANOVA followed by Dunnet’s post hoc test. For each experimental group, Δ% values used for the statistical analysis derived from 4 independent experiments and 50–100 cells were recorded in each different session. (**B**) F (5,1053) = 83.02. * Significant versus control groups and α-syn+Rot+Glut (*p* < 0.0001); ** significant versus all groups (*p* < 0.0001). (**D**) F (5,1245) = 68.75. * Significant versus control groups and α-syn+Rot+Glut (*p* < 0.0001); ** significant versus Ctl, α-syn+Rot, α-syn+Rot+Glut+siEAAT3, α-syn+Rot+siEAAT3 (*p* < 0.0001) and Ctl+siEAAT3 (*p* < 0.001).

**Figure 7:**
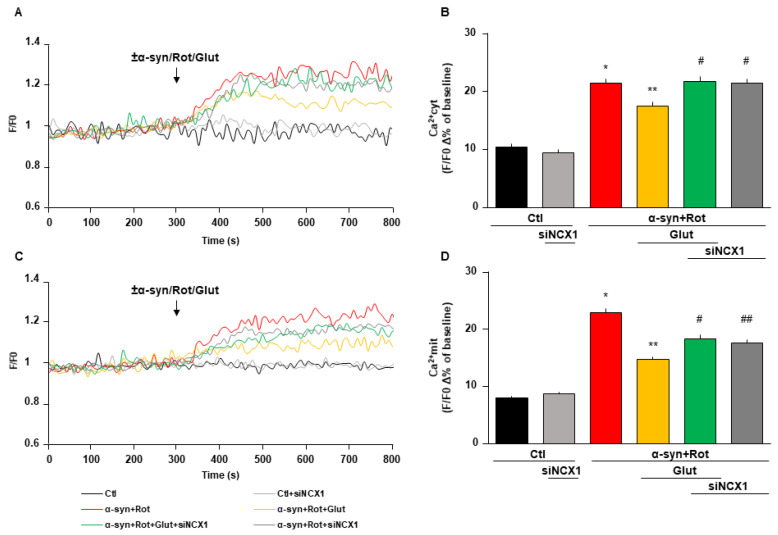
Effect of siNCX1 on glutamate-induced reduction of cytoplasmic and mitochondrial Ca^2+^ levels. (**A**,**C**) Representative records of cytoplasmic (**A**) and mitochondrial (**C**) Ca^2+^ levels under control conditions (black line), after 48 h of NCX1 silencing (light grey line), during acute treatment of α-syn (10 nM) plus Rot (300 nM) in the presence (grey line) or in the absence (red line) of siNCX1, and acute treatment of α-syn plus Rot and glutamate (500 µM) in the presence (green line) or in the absence (orange line) of siNCX1. Fluorescence intensity was expressed as F/F0 ratio, where F is the background subtracted fluorescence intensity and F0 is the background subtracted mean fluorescence value measured from each cell at resting conditions (F/F0). (**B**,**D**) The bar plots showing cytoplasmic (**B**) and mitochondrial (**D**) Ca^2+^ levels depict the mean ± S.E.M of each Δ% fluorescence increase. For Δ% calculation, we used the maximal value of fluorescence obtained after stimulation and, as baseline, the mean of fluorescence recorded during the 300 s preceding the ±α-syn/Rot/Glut challenge. Statistical differences were assessed by one-way ANOVA followed by Dunnet’s post hoc test. (**B**) F (5,1126) = 56.75. For each experimental group, Δ% values used for the statistical analysis derived from at least 4 independent experiments and 50–100 cells were recorded in each different session. * Significant versus control groups (*p* < 0.0001) and α-syn+Rot+Glut (*p* < 0.001); ** significant versus all groups (*p* < 0.0001 versus control groups, α-syn+Rot+Glut+siNCX1 and α-syn+Rot+siNCX1; *p* < 0.001 versus α-syn+Rot); # significant versus control groups and α-syn+Rot+Glut (*p* < 0.0001). (**D**) F (5,1849) = 90.72. For each experimental group, Δ% values used for the statistical analysis derived from at least 5 independent experiments and 50–100 cells were recorded in each different session. * Significant versus all groups (*p* < 0.0001); ** significant versus control groups, α-syn+Rot, α-syn+Rot+Glut+siNCX1 (*p* < 0.0001) and α-syn+Rot+siNCX1 (*p* < 0.01); # significant versus control groups, α-syn+Rot and α-syn+Rot+Glut (*p* < 0.0001); ## significant versus control groups, α-syn+Rot (*p* < 0.0001) and α-syn+Rot+Glut (*p* < 0.01).

When cells were transfected either with EAAT3 or NCX1 siRNA, glutamate failed to prevent the rise of both cytoplasmic and mitochondrial Ca^2+^ levels induced by α-syn plus Rot (Figure 6 and Figure 7).

## 4. Discussion

In the current study, we have shown that, in RA-differentiated SH-SY5Y cells exposed to α-syn plus Rot to reproduce PD-like features, glutamate supplementation significantly improved cell viability by (1) stimulating ATP synthesis, (2) reducing free radical burden, (3) limiting cytosolic and mitochondrial Ca^2+^ overload. Strikingly, glutamate was completely ineffective when either EAAT3 or NCX1 expression was knocked down, disclosing an essential contribution of these transporters in mediating the effect of glutamate on mitochondrial activities. These results highlight the central role of mitochondrial dysfunctions in the pathogenesis of PD and put forward the view that progressive mitochondrial alterations can originate from bioenergetics impairments, and significantly contribute to neuronal death. On this basis, the enhancement of mitochondrial functions appears as a potential strategy to prevent, delay, or reverse PD neurodegenerative processes.

Mitochondrial activities are key determinants of cell functions, since these organelles interface with a plethora of fundamental processes, such as energy production, intracellular redox balance and cellular Ca^2+^ homeostasis [65,66]. Since mitochondrial dynamics are closely integrated, any alteration taking place in one mitochondrial process can profoundly influence other functions, and therewith incite a destructive spiral of energetic failure, oxidative damage and Ca^2+^ overload [12]. Several reports support the pathological relevance of this framework for the neurodegenerative processes underlying PD. For instance, postmortem studies in PD patients have consistently documented a reduction of mitochondrial complex I activity in the nigrostriatal dopaminergic neurons [20,21,67,68,69], as well as a decrease in ATP synthesis, massive ROS production and the dysregulation of Ca^2+^ homeostasis [69,70]. Similar effects can also be ascribed to α-syn and Rot exposure, which can cause a deficit of mitochondrial complex I activity as an upstream event [3,25,64,71,72,73].

Intriguingly, it has been reported that the impairment of complex I activity could be compensated by supplying different substrates, including amino acids (such as glutamate, arginine, proline, valine, aspartate, lysine and glutamine), TCA cycle intermediates (oxaloacetate, citrate and malate) [35], and nutritional supplements like D-β-hydroxybutyrate [34], a membrane-permeable prodrug of succinate [37], or by modulating glutamate metabolism via glutamate dehydrogenase activation [36]. On this basis, we hypothesized that approaches aimed at compensating complex I deficiency may preserve the whole mitochondrial activity and positively impact cell survival in our PD model. In this regard, we focused our attention on glutamate supplementation. Under pathophysiological conditions, glutamate can be used as a metabolic substrate through its conversion to α-ketoglutarate, thereby promoting de novo ATP synthesis by entering the TCA cycle [38,39,40,41]. Here we show that, in RA-differentiated SH-SY5Y cells exposed to α-syn plus Rot, glutamate supplementation promoted cell survival. To explore the mechanisms underlying this positive effect of glutamate, we investigated some critical potentialities of this substrate. First, we verified its metabolic properties and found that, under control conditions, 1 h exposure to glutamate produced a significant increase in ATP synthesis, confirming previous findings obtained in different cell models [38] and in brain and heart isolated mitochondria [41]. When glutamate was added during the last hour of the treatment, the drop in ATP content induced by α-syn plus Rot was completely reverted. Moreover, the finding that oligomycin, an inhibitor of ATP synthase, abolished such recovery indicated that the increase in ATP synthesis induced by glutamate mainly derived from an ex novo process, which used glutamate as the main substrate.

Within inner mitochondrial membrane, complex I works in parallel with complex II, which transfers electrons from FADH_2_ produced during succinate oxidation [74]. Although the contribution of this pathway to the oxidative phosphorylation is normally minimal, we hypothesized that, when complex I is compromised, glutamate can still feed the TCA cycle at the level of α-ketoglutarate, allowing dopaminergic cells to circumvent complex I and directly providing electrons from FADH_2_ to complex II, finally increasing ATP generation.

Since glutamate can also participate in the synthesis of antioxidant molecules [43,75,76], we explored whether the observed protective effects could have also derived from this property. By assessing mitochondrial ROS levels, we found that, as expected, they were dramatically increased as a consequence of α-syn plus Rot treatment, and that the exposure to glutamate significantly reduced such an increase. In addition, in view of the main role of mitochondria in controlling Ca^2+^ homeostasis, we also sought to explore the Ca^2+^ dynamics taking place in our experimental setting. We found that the acute exposure to α-syn plus Rot induced both cytoplasmic and mitochondrial Ca^2+^ alterations, which were significantly contained by glutamate addition. Overall, mitochondrial bioenergetic deficits and an increased mitochondrial oxidative damage coupled with a dysregulation of Ca^2+^ homeostasis sensitized cells to intrinsic death programs, which were halted by glutamate. We did not explore the exact sequence that characterized these events. However, on the basis of the available literature, we tentatively speculate that, on the one hand, complex I alteration may trigger a bioenergetics crisis, and, on the other hand, it may favor electron leakage from the transport chain into the mitochondrial matrix, causing ROS overproduction [14,77,78]. These events initiate a vicious cycle, which may give rise to a persistent loss of both cytosolic and mitochondrial membrane potential, leading to a massive Ca^2+^ influx [14,77], which in turn may fuel ROS production and ATP loss [79]. In this framework, glutamate would act by preserving the whole mitochondrial functions: by restoring energy metabolism and by improving the antioxidant defenses of the cell, it protects mitochondria from oxidative damage and ameliorates their bioenergetics performance at the same time.

Another interesting finding emerging from our study is that glutamate effect occurred in an EAAT3/NCX1-dependent fashion. We found that the protective actions of glutamate were completely lost after both pharmacological inhibition and RNA silencing of either EAAT3 or NCX1. Although in our experimental conditions RA-differentiated SH-SY5Y cells express also EAAT1-2 (data not shown) and NCX3 [40], we focused on EAAT3 and NCX1 because the molecular and functional coupling of these specific transporters has been already observed at both the plasma membrane and mitochondrial levels in different models under pathophysiological conditions [38,39,40,41]. Although the intracellular distribution of EAAT3 is less characterized than its surface membrane expression [80,81,82], being still open to further analysis, it is worth noting here that different research groups provided evidence for the localization of glutamate transporters from the EAAT family also in the mitochondrial compartment [83,84,85,86].

EAAT3/NCX1 colocalization underlies a mechanism where the entry of glutamate into the cells and mitochondria would favor the reverse mode activity of NCX, because of the increase in Na^+^ levels that accompany the EAAT-dependent uptake of glutamate. In this way, Ca^2+^ levels are influenced as well. In line with this observation, in control conditions, glutamate can induce a slight but significant increase in both cytosolic and mitochondrial Ca^2+^ levels (Figure 5), consistently with our previous findings [38,41] and as already proposed in astrocytes [87,88]. The latter event activates the mitochondrial Ca^2+^ sensitive dehydrogenases, driving glutamate utilization and enhancing ATP synthesis [50]. It is worth noting that both Ca^2+^ and glutamate are essential to promote the synthesis of ATP [38]. Interesting, we have previously shown that NCX3 does not support such a mechanism, since its specific silencing does not compromise glutamate-induced neuroprotection [40]. Second, a primary role of EAAT3 for the metabolic use of glutamate to fuel energetics has been already proposed in dopaminergic neurons [89]. Overall, this machinery ensures an efficient glutamate uptake, which preserves both cellular bioenergetics and antioxidant defenses. Therefore, a deficiency in their activities may compromise the intracellular utilization of glutamate, causing the loss of its protective actions. The results of the present study further support the concept that a functional interplay between these transporters is a basic requirement for proper functioning of the “glutamate machinery”, otherwise known as “glutamosome” [43,84]. Furthermore, it is worth noting that, on the one hand, NCX1 knock-down may be negative since it prevented glutamate utilization; on the other hand, it may be protective against mitochondrial Ca^2+^ overload in the absence of glutamate [22]. This apparent discrepancy underlies a dual role of NCX1, which operates in different ways depending on the specific molecular environment, as already observed in other pathological settings [40,90].

Experimental settings used in our work to explore and characterize the potential of glutamate-driven metabolism to limit PD-like degeneration were chosen to minimize excitotoxic neuronal damage, as discussed below. In our model glutamate, in terms of both concentration and time of exposure, had no evident toxic effects under control conditions, which is consistent with previous studies based on SH-SY5Y cells, where higher concentrations of glutamate (up to tens of millimolar levels) and/or longer exposure (within hours-to-days time frames) were reported to significantly impair cell functions/viability [91,92,93,94,95]. Several lines of evidence converge to support that the metabolic fate of glutamate in brain cells is highly influenced by the glutamate levels in the extracellular milieu: glutamate metabolism that feeds ATP-generating pathways is typically favored when neuronal (including mesencephalic cell preparations) or glial cultures are exposed for 1–2 h to glutamate concentrations in the hundreds of micromolar range [40,89,96,97,98]. Similar levels can be transiently reached following glutamate release within extracellular compartments at the tripartite synapse structures [99,100] and do not significantly affect viability when primary neuronal, glial or cardiac cell cultures are similarly exposed to glutamate for 1–3 h [39,89,96,101,102]. Although in our model EAAT/NCX transporters emerge as a protective gateway that contributes to cellular uptake and metabolism of glutamate, three major complexities remain to be further addressed. First, with the intent to move from in vitro to in vivo studies, the characterization of the EAAT/NCX pathway for glutamate use in PD cannot simply rely on exogenous glutamate administration, since this amino acid does not easily cross an intact blood–brain barrier [99,103] and/or can provoke undesirable excitotoxic insults [42,104]. To this end, an approach aimed at modulating EAAT/NCX functional interaction would be more feasible to safely influence both extracellular glutamate levels and its intracellular metabolic use. It is worth mentioning here that other interventions that similarly favor glutamate metabolism were shown to improve cell bioenergetics as well as promote clearance of extracellular glutamate and survival of degenerating neurons after an ischemic insult [105,106]. Second, we cannot rule out a possible involvement of metabotropic receptors (mGluRs) for the glutamate benefits observed in our models, since mGluRs have been detected in RA-differentiated SH-SY5Y cells [91,107]. Third, the relevance of EAAT/NCX functional interaction in PD pathology needs to be also explored in glial cells, which normally contribute to the maintenance of brain homeostasis [108], can metabolically respond to glutamate in an EAAT/NCX-dependent manner [38,41] and ultimately can play a relevant role in degenerative disorders, including PD [109,110,111].

Collectively, results from the present study add further ground to the hypothesis that mitochondrial dysfunctions are major drivers of the neurodegenerative cascade underlying dopaminergic neuronal death in PD. The modulation of EAAT/NCX function may be an effective step forward in tackling the challenge of PD neurodegeneration, since it has the potential of simultaneously address both glutamate-mediated excitotoxicity (by favoring cell glutamate uptake) and the upstream energetic failure (by favoring glutamate metabolic use) that incite the progression of PD pathology. In this regard, further studies are required to understand the specific role of EAAT3-NCX1 functions in contributing to glutamate metabolic use in animal models of PD.
